# Diversity and functions of the sheep faecal microbiota: a multi‐omic characterization

**DOI:** 10.1111/1751-7915.12462

**Published:** 2017-02-06

**Authors:** Alessandro Tanca, Cristina Fraumene, Valeria Manghina, Antonio Palomba, Marcello Abbondio, Massimo Deligios, Daniela Pagnozzi, Maria Filippa Addis, Sergio Uzzau

**Affiliations:** ^1^ Porto Conte Ricerche, Science and Technology Park of SardiniaTramariglioAlgheroItaly; ^2^ Department of Biomedical SciencesUniversity of SassariSassariItaly

## Abstract

Little is currently known on the microbial populations colonizing the sheep large intestine, despite their expected key role in host metabolism, physiology and immunity. This study reports the first characterization of the sheep faecal microbiota composition and functions, obtained through the application of a multi‐omic strategy. An optimized protocol was first devised for DNA extraction and amplification from sheep stool samples. Then, 16S rDNA sequencing, shotgun metagenomics and shotgun metaproteomics were applied to unravel taxonomy, genetic potential and actively expressed functions and pathways respectively. Under a taxonomic perspective, the sheep faecal microbiota appeared globally comparable to that of other ruminants, with Firmicutes being the main phylum. In functional terms, we detected 2097 gene and 441 protein families, finding that the sheep faecal microbiota was primarily involved in catabolism. We investigated carbohydrate transport and degradation activities and identified phylum‐specific pathways, such as methanogenesis for Euryarchaeota and acetogenesis for Firmicutes. Furthermore, our approach enabled the identification of proteins expressed by the eukaryotic component of the microbiota. Taken together, these findings unveil structure and role of the distal gut microbiota in sheep, and open the way to further studies aimed at elucidating its connections with management and dietary variables in sheep farming.

## Introduction

Sheep farming is widespread worldwide for the purpose of meat, milk, skin and/or wool production. According to the specific productive purpose, farmers select sheep breeds and, traditionally, efforts are continuously made to improve physical/genetic traits that, in turn, ameliorate the production performances.

Regardless of the genetic background and of the production purpose, a correct nutritional management plays a crucial role in warranting healthy and fertile sheep and productive dairy ewes. Sheep are grazers and eat a variety of plants, including grass, clover and weeds. In addition to pasture grazing, sheep are generally fed with hay and a controlled amount of grain. As for other dairy ruminants, energy stored in plant matter is eventually converted to protein food products (i.e., milk and meat) after a complex digestive process. The plant mass is first fermented by the ruminal microbial communities, and a large part of the organic matter is then degraded and partially adsorbed in the sheep four‐chambered stomach. Bacteria involved in this process have a widest range of biochemical activities enabling digestion of cellulose, hemicellulose, starch and proteins. While storage polysaccharides (starch) can be promptly degraded in the rumen ecosystem, full degradation of structural polysaccharides (i.e. cellulose) and resistant starch is a longer and more complex process (Huntington, 1997; Krause *et al*., 2003). In addition, a number of ruminal bacteria have been described to be involved in the isomerization and saturation of the dietary unsaturated fatty acids, leading to the sheep milk saturated fat composition (Huws *et al*., 2011). Short‐chain fatty acids (SCFAs) are also produced as a consequence of carbohydrate fermentation. Further, rumen microorganisms and food residues pass on to the abomasum and the small intestine where food degradation endures by microbial and host‐secreted enzymes. Finally, undigested organic matter reaches the large intestine where it undergoes the last digestive processes by the colonic microbial population, before water and salt absorption by colonic mucosa completes the formation of the faecal pellets. Here, degradation of both resistant starch and fibres is expected to occur, as in monogastric vertebrates colon, as well as SCFA production and absorption by the colonic mucosa. A considerable amount of data have been collected on the composition and functions of sheep rumen microbiota (Shi *et al*., 2014; Brilhante *et al*., 2015; Morgavi *et al*., 2015; Zeng *et al*., 2015), but much less is currently known on the microbial populations that colonize the large intestine, despite their crucial role in the sheep intestinal metabolism.

As in other non‐ruminant mammalian, including humans, the microbial population that colonizes the sheep large intestine is expected to be key in providing energy, antigens and metabolites that positively affect host metabolism, physiology and immunity. Basically, a well‐balanced microbiota, with highly diverse taxonomic content and stability, appears of paramount importance throughout the whole digestive system, where a bidirectional driving force between microbial metabolic circuits and mucosal physiology allows to maintain a stable microbiota and a healthy gut and, consequently, an overall healthy and productive organism.

In keeping with these premises, we investigated composition and functions of the microbial populations associated with the final tract of the sheep large intestine, where the last stage of plant mass digestion occurs with a significant potential contribution to host energy harvesting and physiology homeostasis. To reach this aim, we employed an integrated, multi‐omic strategy, comprising 16S rDNA and shotgun metagenomic sequencing, to unravel microbiota structure and genetic potential, as well as metaproteomics, to identify and characterize functions and pathways actively expressed by the sheep faecal microbial communities.

## Results and discussion

### Optimization of protocols for DNA extraction and sample clean‐up for the analysis of the sheep faecal microbiota

Stool is a complex sample matrix with regard to DNA extraction. Sample pretreatment protocols, such as differential centrifugation (DC), have been reported to help bacterial DNA extraction from the faecal matrix (Apajalahti *et al*., 1998), although direct lysis (DL) of the microbial matter by chemical or mechanical methods (or a combination of these) is the most widely applied strategy (Hart *et al*., 2015; Wagner Mackenzie *et al*., 2015). Several substances may be co‐extracted having inhibitory effects on downstream analysis, with even massive influence on quality of the extracted DNA, and consequently on feasibility and outcome of DNA sequencing. This is especially key when dealing with stool from sheep, whose distal colon reduces the faecal water content up to 65% (Hecker and Grovum, 1975). Here, we compared five different sample preparation/extraction methods from sheep faecal samples and used 16S rRNA gene amplification efficiency as a probe to determine the DNA suitability for downstream metagenomic analysis. Specifically, as described in the “Experimental procedures” section, samples were subjected to DC or DL; then, DNA was extracted from both preparations after enzymatic and mechanical lysis with QIAamp Fast DNA Stool or with the E.Z.N.A. Soil DNA Kit. Finally, samples were also subjected to DC and then extracted with the standard phenol/chloroform/isoamyl alcohol (25:24:1) method.

As a result, the combination of stool DC preparation followed by DNA extraction and purification with the E.Z.N.A. soil DNA kit, designed to remove with highest efficiency PCR inhibitors, was the only protocol capable of providing a satisfactory quantity (25 ± 0.28 ng g^−1^ stool sample) and the best quality of extracted DNA (100% of samples providing a 16S rRNA gene PCR amplification product; data not shown).

### Sheep faecal microbiota composition

To assess the faecal microbiota composition in sheep, we analysed its metagenome, by sequencing the V4 region of the 16S rDNA (V4‐MG) and the whole microbial DNA (shotgun metagenomics, S‐MG), as well as its metaproteome (MP), by means of shotgun mass spectrometry. These were intended as complementary approaches, being based on the measurement of different molecules (DNA for V4‐MG and S‐MG, peptides for MP) with different strategies (targeted for V4‐MG, shotgun for S‐MG and MP). The whole DNA sequencing and peptide mass spectrometry metrics are presented in Table S1. Read counts (for MG approaches) and spectral counts (for MP) were used throughout the study to estimate the relative abundance of the taxonomic and functional features of the microbiota.

**Figure 1:**
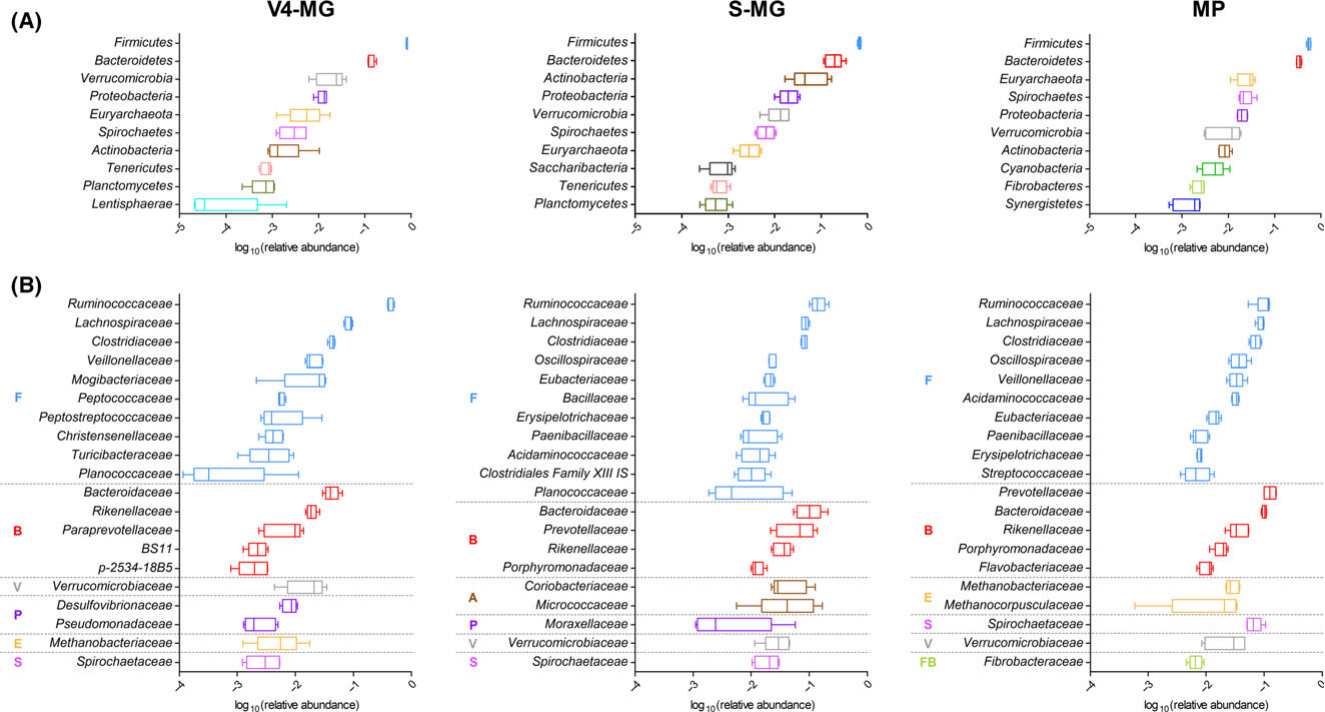
Taxonomic composition of the sheep faecal prokaryotic microbiota, according to V4‐16S rRNA (V4‐MG, left), metagenomic (S‐MG, centre) and metaproteomic (MP, right) results. (A) Tukey's box plot illustrating the microbiota composition at phylum level. The top 10 phyla are shown, ordered by decreasing mean relative abundance. (B) Tukey's box plot illustrating the microbiota composition at family level. The top 20 families are shown, grouped based on the relative phylum (A, Actinobacteria; B, Bacteroidetes; E, Euryarchaeota; F, Firmicutes; FB, Fibrobacteres; P, Proteobacteria; S, Spirochaetes; V, Verrucomicrobia) and further ordered by decreasing mean relative abundance.

The taxonomic composition of the prokaryotic microbiota according to V4‐MG, S‐MG and MP results is shown in Fig. 1. As expected, Firmicutes and Bacteroidetes made over 80% of total bacteria in all cases. Moreover, Firmicutes was detected as the most represented phylum in all animals and with all approaches, followed by Bacteroidetes (Fig. 1A). However, the average Firmicutes‐to‐Bacteroidetes ratio (F/B) ranged from 6.0 for V4‐MG down to 1.6 for MP, through 3.4 for S‐MG. The archaeal Euryarchaeota was the fifth, seventh and third most abundant phylum according to V4‐MG, S‐MG and MP data respectively. While Firmicutes levels were well conserved among individuals (CV < 10% with all approaches), a higher variation could be observed for other important phyla, especially for Actinobacteria and Verrucomicrobia. When comparing our data with the existing studies reporting a metagenomic analysis of faecal samples from other ruminants, we could find a general predominance of Firmicutes over Bacteroidetes in cattle, with Ruminococcaceae and Lachnospiraceae being the most representative microbial families of the former phylum (Durso *et al*., 2010, 2011; Shanks *et al*., 2011; Kim *et al*., 2014), in line with our results obtained in sheep. Conversely, Bacteroidetes was the most abundant phylum in the sheep rumen according to the literature (Castro‐Carrera *et al*., 2014; de la Fuente *et al*., 2014; Kittelmann *et al*., 2015; Lopes *et al*., 2015; Morgavi *et al*., 2015). Consistently, a marked increase in the F/B ratio from rumen to colon was recently described in cow (Mao *et al*., 2015). Among minor phyla, we found a remarkable amount of functionally active Spirochaetes, mainly belonging to the genus *Treponema*, especially *T. saccharophilum*, early described as a large pectinolytic spirochaete present in the rumen (Paster and Canale‐Parola, 1985). This phylum was recently observed as the fourth most abundant within the microbiota of the ruminant digestive tract (Peng *et al*., 2015).

S‐MG and MP approaches also allowed for the identification of fungal taxa, accounting for about 0.05% and 1.1% of the microbiota respectively. Ascomycota was detected in both cases as the most abundant fungal phylum.

Going down to the family level, 76, 385 and 171 different microbial families were detected with V4‐MG, S‐MG and MP respectively. Considering the ‘core microbiota’ (i.e. taxa found consistently in all animals analysed), V4‐MG, S‐MG and MP analyses led to find a total of 45, 168 and 50 microbial families, respectively, of which 19 in common between the three approaches. As shown in Fig. 1B, Ruminococcaceae, Lachnospiraceae and Clostridiaceae were consistently found with all approaches to be the first, second and third most abundant family within Firmicutes, respectively, consistent with previous studies in cows (Durso *et al*., 2010, 2011; Shanks *et al*., 2011; Kim *et al*., 2014). On the other hand, bacterial families belonging to Bacteroidetes exhibited more variable distributions, with Prevotellaceae becoming the main family overall when considering protein expression data. Families from other phyla, such as Spirochaetaceae from Spirochaetes and Verrucomicrobiaceae from Verrucomicrobia, were also found among the top 20 abundant families with all techniques.

Based on our results, V4‐MG, S‐MG and MP provided generally comparable taxonomic distributions, with some slight but important differences, such as the F/B ratio and the relative abundance of Prevotellaceae. This is largely expected when dealing with conceptually and technically different approaches, as previously outlined. In addition, microbial taxa bearing a higher number of 16S rRNA genes are expected to be overestimated when compared with those with a lower number according to V4‐MG analysis (Vetrovsky and Baldrian, 2013), and species with smaller genome size are expected to be underestimated when compared with those with larger size according to S‐MG analysis (Nayfach and Pollard, 2015). As a confirmation, in this work, when comparing V4‐MG and S‐MG results, phyla with larger genomes on average (i.e. Actinobacteria and Proteobacteria) reached a higher rank in abundance with S‐MG, while those with smaller genomes (i.e. Verrucomicrobia and Tenericutes) were more represented with V4‐MG. In addition, it is worth noting that some data analysis‐related biases may considerably influence comparability of outputs, such as differences in taxonomic classification and update frequency among databases (GreenGenes versus NCBI).

The complete taxonomic distribution data (also comprising class, order and genus levels) for V4‐MG, S‐MG and MP are presented in Data S1–S3.

### Assessment of functions potentially and actively expressed by the sheep faecal microbiota

**Figure 2:**
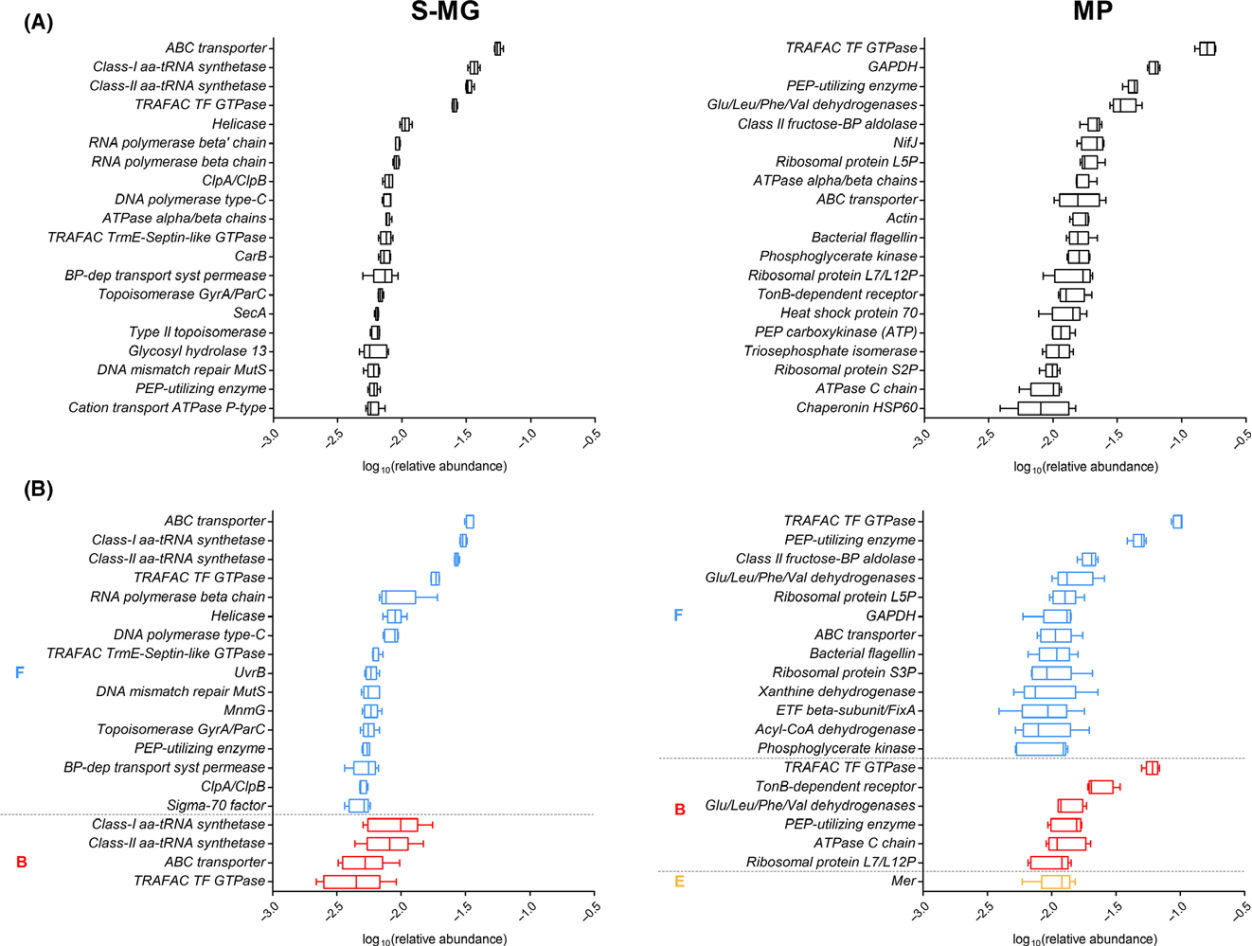
Functional potential and activity of the sheep faecal microbiota, as measured by metagenomics (S‐MG, left) and metaproteomics (MP, right) respectively. (A) Tukey's box plot illustrating the 20 most abundant gene (left) and protein families. (B) Tukey's box plot illustrating the 20 most abundant gene family–phylum (left) and protein family–phylum (right) combinations, grouped based on the relative phylum (B, Bacteroidetes; E, Euryarchaeota; F, Firmicutes) and further ordered by decreasing mean relative abundance.

Figure 2A illustrates the 20 most abundant gene and protein families found upon S‐MG and MP analyses respectively. As explained above, the former data regard the functional potential of the microbiota, while the latter represent the key functions actually exerted by the faecal microorganisms. The metagenome was found to be rich in genes related to membrane transport of molecules (ABC transporter, ATPase, permease, SecA), DNA replication and repair (helicase, DNA polymerase, topoisomerase, MutS), transcription (RNA polymerase), translation (tRNA synthetases and translation factors) and protein folding (chaperones), plus a few encoding for metabolic enzymes. As revealed by MP data, among the most abundant protein families expressed by the faecal microbiota, we primarily found functions related to metabolism (eight enzymes), especially carbohydrate degradation, followed by protein synthesis and folding (translation factor, ribosomal proteins, chaperones). Protein families involved in transport and signalling, such as ABC transporters, TonB‐dependent receptor, ATPases, flagellin, were also present. On the whole, 2097 gene families and 441 protein families were identified by S‐MG and MP respectively. Considering the ‘core functions’ (i.e. associated with the microbiota of all the animals analysed), S‐MG and MP analyses led to detect a total of 904 and 191 functional families, respectively, of which 152 were identified both as genetic trait and as expressed proteins (‘core’ protein families are listed in Table S2).

When considering function–taxonomy combinations at the phylum level (Fig. 2B), consistently with the sole taxonomic information, we found a higher representation of functions encoded/expressed by Firmicutes, compared with those encoded/expressed by Bacteroidetes. According to MP data, some functions from other phyla (namely from Euryarchaeota) were also considerably expressed. More interestingly, the most represented genes assigned to Firmicutes according to S‐MG results were in most cases identical to the most represented genes assigned to Bacteroidetes, whereas, when considering the main expressed protein functions, several of them were not overlapping between these two phyla. To further investigate this ‘phylum‐specific’ functional contribution, we sought for those protein families exclusively and unambiguously assigned to a single phylum, and detected in all samples (42 in total). As shown in Table 1, several relevant and considerably abundant protein functions were actually phylum specific, whereas the 60 phylum‐specific ‘core’ genes were all detected at very low abundance (Table S3). Among protein functions, we detected the TonB‐dependent receptor as specific for Bacteroidetes, the enzyme 5,10‐methylenetetrahydromethanopterin reductase (Mer) for Euryarchaeota, involved in methanogenesis, as well as aldehyde oxidoreductase (belonging to the xanthine dehydrogenase family), formate‐tetrahydrofolate ligase and carbon monoxide dehydrogenase (both involved in one‐carbon metabolism) for Firmicutes.

**Table 1:** Protein families assigned exclusively to a single phylum and detected in all samples, ordered by phylum and then by mean percentage abundance

Protein family	Phylum	Sheep 1	Sheep 2	Sheep 3	Sheep 4	Sheep 5	Mean
TonB‐dependent receptor	Bacteroidetes	2.021%	2.036%	1.912%	2.628%	3.400%	2.399%
Group II decarboxylase	Bacteroidetes	0.218%	0.375%	0.421%	0.113%	0.174%	0.260%
Ribosomal protein S1P	Bacteroidetes	0.273%	0.402%	0.153%	0.188%	0.262%	0.255%
NagA	Bacteroidetes	0.109%	0.161%	0.268%	0.188%	0.087%	0.163%
GHMP kinase	Bacteroidetes	0.055%	0.161%	0.115%	0.075%	0.174%	0.116%
ExbB/TolQ	Bacteroidetes	0.164%	0.054%	0.153%	0.038%	0.131%	0.108%
Class‐I fumarase	Bacteroidetes	0.164%	0.080%	0.038%	0.075%	0.087%	0.089%
Gfo/Idh/MocA	Bacteroidetes	0.055%	0.054%	0.038%	0.038%	0.131%	0.063%
Eukaryotic mitochondrial porin	Basidiomycota	0.055%	0.027%	0.076%	0.038%	0.044%	0.048%
RuBisCO large chain	Cyanobacteria	0.218%	0.054%	0.191%	0.300%	0.305%	0.214%
Reaction centre PufL/M/PsbA/D	Cyanobacteria	0.055%	0.080%	0.115%	0.113%	0.044%	0.081%
Mer	Euryarchaeota	1.202%	0.589%	1.262%	1.201%	1.526%	1.156%
[NiFe]/[NiFeSe] hydrogenase large subunit	Euryarchaeota	0.546%	0.054%	0.574%	0.488%	1.133%	0.559%
MTD	Euryarchaeota	0.164%	0.080%	0.115%	0.225%	0.305%	0.178%
MtrA	Euryarchaeota	0.164%	0.080%	0.229%	0.150%	0.262%	0.177%
FrhB	Euryarchaeota	0.164%	0.027%	0.115%	0.150%	0.218%	0.135%
Archaeal histone HMF	Euryarchaeota	0.109%	0.107%	0.038%	0.075%	0.087%	0.083%
Ribosomal protein L12P	Euryarchaeota	0.055%	0.027%	0.076%	0.075%	0.131%	0.073%
N‐Me‐Phe pilin	Fibrobacteres	0.055%	0.027%	0.038%	0.113%	0.087%	0.064%
Xanthine dehydrogenase	Firmicutes	2.294%	0.509%	1.033%	0.751%	0.741%	1.065%
Formate–tetrahydrofolate ligase	Firmicutes	0.874%	0.589%	0.727%	0.526%	0.567%	0.656%
Ni‐containing carbon monoxide dehydrogenase	Firmicutes	0.492%	0.509%	0.727%	0.563%	0.349%	0.528%
ETF alpha subunit/FixB	Firmicutes	0.492%	0.456%	0.421%	0.638%	0.262%	0.453%
Complex I 51 kDa subunit	Firmicutes	0.328%	0.643%	0.459%	0.263%	0.305%	0.400%
FldB/FldC dehydratase beta subunit	Firmicutes	0.819%	0.295%	0.306%	0.413%	0.044%	0.375%
Acetyl‐CoA hydrolase/transferase	Firmicutes	0.328%	0.241%	0.306%	0.263%	0.218%	0.271%
Elongation factor P	Firmicutes	0.109%	0.241%	0.268%	0.188%	0.392%	0.240%
Diol/glycerol dehydratase small subunit	Firmicutes	0.273%	0.080%	0.153%	0.263%	0.305%	0.215%
Glycosyltransferase 1	Firmicutes	0.109%	0.161%	0.268%	0.150%	0.131%	0.164%
Glycosyl hydrolase 101	Firmicutes	0.055%	0.161%	0.229%	0.150%	0.044%	0.128%
Glyoxalase I	Firmicutes	0.164%	0.080%	0.153%	0.150%	0.087%	0.127%
Bacterial solute‐binding protein 1	Firmicutes	0.164%	0.107%	0.153%	0.075%	0.131%	0.126%
Acyl‐CoA mutase large subunit	Firmicutes	0.109%	0.107%	0.115%	0.113%	0.131%	0.115%
Diol/glycerol dehydratase medium subunit	Firmicutes	0.109%	0.080%	0.076%	0.150%	0.131%	0.109%
V‐ATPase proteolipid subunit	Firmicutes	0.164%	0.080%	0.153%	0.038%	0.087%	0.104%
Glutamine synthetase	Firmicutes	0.055%	0.080%	0.076%	0.075%	0.131%	0.083%
Aldolase class II	Firmicutes	0.055%	0.134%	0.115%	0.038%	0.044%	0.077%
GSP E	Firmicutes	0.055%	0.027%	0.038%	0.038%	0.044%	0.040%
Hfq	Firmicutes	0.055%	0.027%	0.038%	0.038%	0.044%	0.040%
Peptidase S41A	Planctomycetes	0.055%	0.027%	0.038%	0.075%	0.044%	0.048%
Resistance–nodulation–cell division	Proteobacteria	0.055%	0.027%	0.038%	0.038%	0.044%	0.040%

Among other expressed protein functions of possible interest, we can cite flagellins, which mainly belonged to Firmicutes (from genera such as *Clostridium* and *Selenomonas*) and Spirochaetes (again, essentially *Treponema*). Notably, half of the functionally annotated Spirochaetes peptides identified in this study were from flagellar proteins. In fact, members of this phylum are known to have long flagella, enclosed in the periplasm and capable to confer them a unique motility (Wolgemuth, 2015). Moreover, we were able to identify SASPs (small, acid‐soluble spore proteins), mainly of clostridial origin, in four of five samples, indicating the presence of endospores within the faecal microbiota of these animals.

As a further consideration, we note that the massive discrepancy found between S‐MG and MP functional results, which should be mainly attributed to actual (and largely expected) differences between genetic potential and protein expression, may also be partially due to varying sample pretreatment strategies. As stated above, DNA extraction required DC steps to ensure efficient PCR amplification, while protein extraction was performed on the faecal material as is to avoid artifactual depletion of food‐bound microbes (Tanca *et al*., 2015).

Complete data concerning gene and protein families identified upon S‐MG and MP analyses are presented in Data S2 and S3 respectively.

### Microbial metabolic pathways in sheep gut

**Figure 3:**
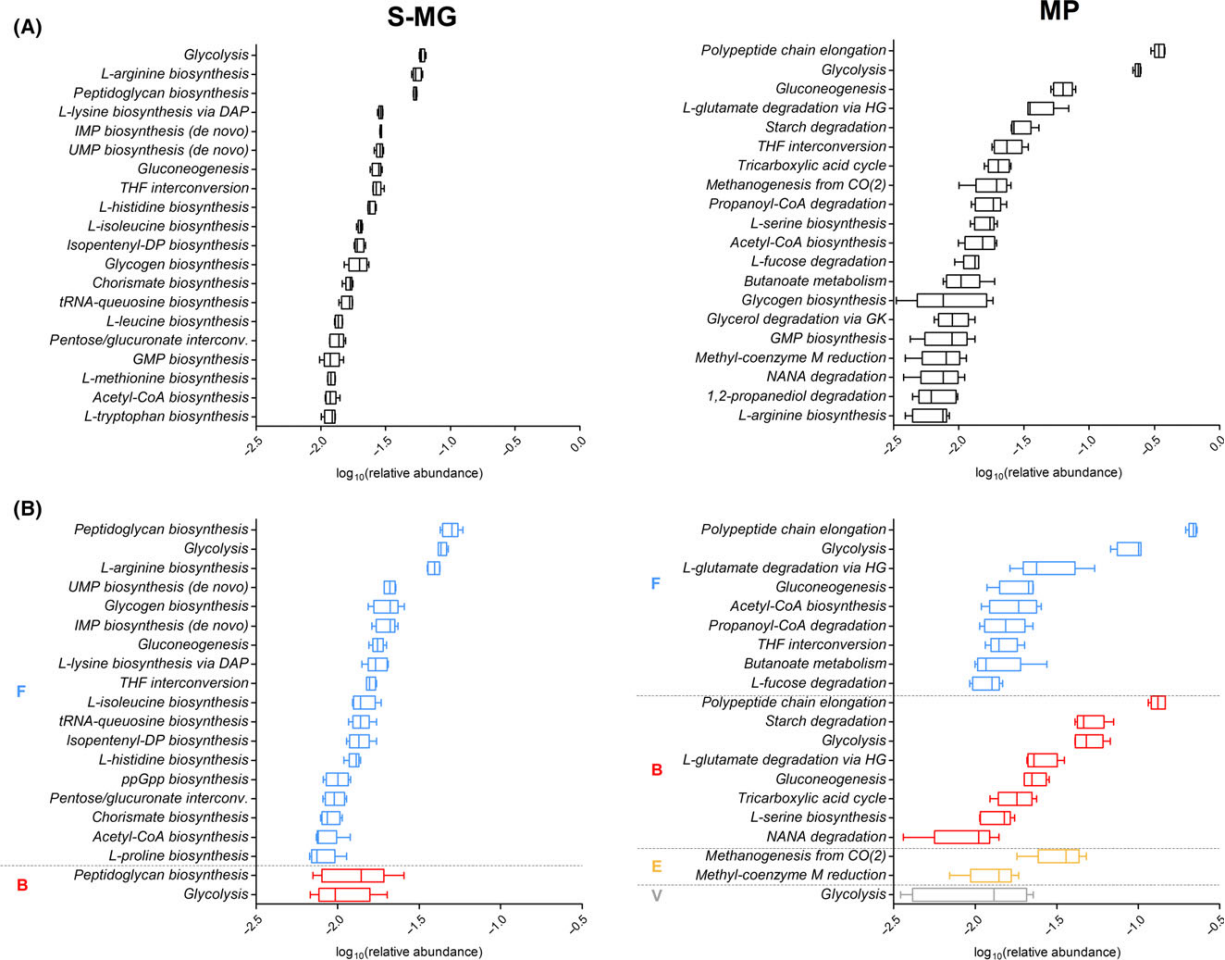
Metabolic pathway potential and activity of the sheep faecal microbiota, as measured by metagenomics (S‐MG, left) and metaproteomics (MP, right) respectively. (A) Tukey's box plot illustrating the 20 most relevant pathways, based on the related gene (left) and protein (right) abundance. (B) Tukey's box plot illustrating the 20 most relevant pathway–phylum combinations, based on the related gene (left) and protein (right) abundance. Pathways are grouped based on the relative phylum (B, Bacteroidetes; E, Euryarchaeota; F, Firmicutes; V, Verrucomicrobia) and further ordered by decreasing mean relative abundance.

Gene and protein functional data were further grouped based on the UniProt ‘pathway’ annotation. Figure 3A illustrates the main metabolic pathways potentially and actively functioning in the sheep faecal microbiota, according to S‐MG and MP data respectively. Several amino acid, nucleoside and carbohydrate biosynthetic routes were consistently highly represented both in the metagenome and in the metaproteome. However, while most of the main genes‐according to S‐MG data‐ were related to biosynthetic pathways, MP data revealed catabolic activities of the microbiota as clearly prominent in terms of active expression and relative abundance. Furthermore, enzymes involved in methanogenesis were considerably more abundant than expected, considering their gene content as assessed by S‐MG analysis.

Figure 3B illustrates pathway–taxonomy combinations at phylum level, revealing that, although the large majority of pathways covered in the metagenome were related to Firmicutes, several different phyla do actually participate in the metabolism at comparable extents, according to MP results. Phylum‐specific ‘core’ pathways could also be identified, namely 1,2‐propanediol degradation and butanoate metabolism for Firmicutes, starch degradation for Bacteroidetes and methanogenesis for Euryarchaeota. Among them, utilization of 1,2‐propanediol is usually mediated by a bacterial microcompartment, in which a multiprotein shell encapsulates enzymes and cofactors for 1,2‐propanediol catabolism, sequestering the reactive propionaldehyde to limit its cellular toxicity (Havemann *et al*., 2002). We found considerable amounts of propanediol utilization protein (PduA) mainly produced by members of Clostridia, in line with recent evidences correlating fucose and rhamnose metabolism with propanediol utilization microcompartments in *Clostridium phytofermentans* (Petit *et al*., 2013). Furthermore, production of butyric acid by specific members of Firmicutes has been largely demonstrated as a key process in the host–microbiota cross‐talk in mammalians, likely related to intestinal health (Pryde *et al*., 2002; Hamer *et al*., 2009; Vital *et al*., 2014).

**Figure 4:**
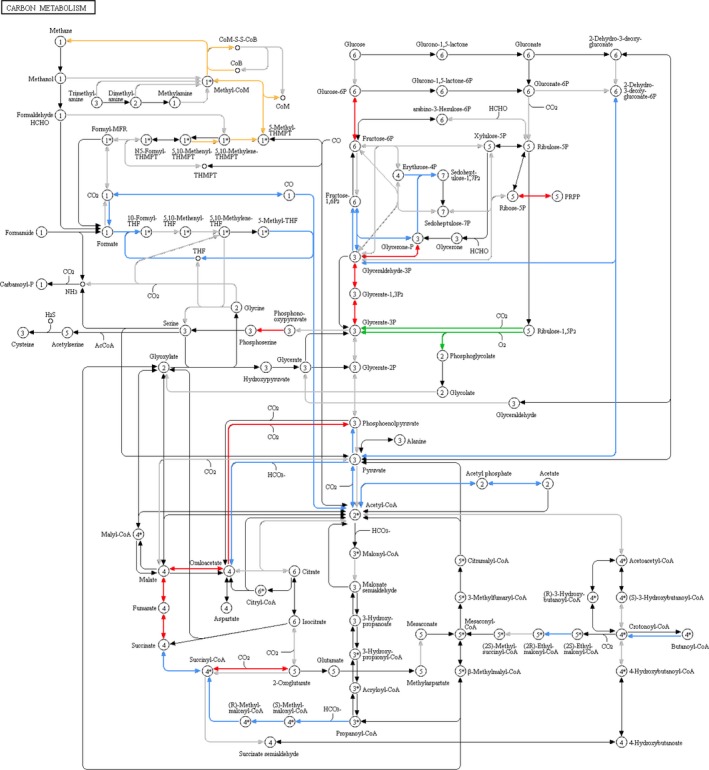
Enzymatic functions identified by metaproteomics and mapped in the KEGG carbon metabolism pathway. Coloured arrows indicate enzymes detected in all animals, with the colour corresponding to the main phylum to which the function was assigned (red, Bacteroidetes; blue, Firmicutes; orange, Euryarchaeota; green, Cyanobacteria). Grey arrows indicate enzymes detected in at least one but not all animals, or not assigned unambiguously to at least one phylum.

We then focused our attention specifically on carbon metabolism and mapped the identified proteins into the corresponding KEGG pathway in order to investigate which microbial players were mainly involved in each specific enzymatic step. While glycolytic reactions were revealed to be carried out in parallel by several different phyla, Fig. 4 clearly illustrates how other metabolic functions are exerted in a phylum‐specific fashion within the sheep faecal microbiota. First, these data evidence, as expected, the unique contribution of Archaea to methanogenesis. Characterization and monitoring of methanogenesis in ruminants is receiving growing attention for zootechnical, environmental and ecological reasons (Kumar *et al*., 2014; Shi *et al*., 2014). Moreover, activity of methanogens in lambs’ caecum, but not in the rumen, has been reported to be affected by specific diets, suggesting a possible compensation of rumen methane production with caecum methanogenesis (Popova *et al*., 2013). In this work, while S‐MG led to detect a minimum amount of archaeal genes, of which none directly involved in methanogenic reactions, MP allowed us to reconstruct almost entirely the methanogenic route (from 5,10‐methenyltetrahydromethanopterin to methane, including those enzymes responsible for methyl‐coenzyme M reduction) in the microbiota of all animals analysed, and to assign the corresponding enzymatic functions to members of Methanobacteriaceae and Methanocorpusculaceae families, specifically *Methanobrevibacter ruminantium* and *Methanocorpusculum labreanum* respectively. This might be due to a massively higher amount of enzymes synthesized when compared with the number of archaeal genes and/or cells, as well as to greater difficulties in co‐extraction of archaeal DNA compared with proteins. It is also important to note that the presence of publicly available archaeal sequences of methanogenic genes within the database used for MP analysis compensated the absence of experimental metagenomic sequences and allowed for an efficient peptide identification. Furthermore, these results confirm the ability of metaproteomics to map enzyme expression for entire pathways, as already described in the case of methanogens (Kohrs *et al*., 2014; Gunnigle *et al*., 2015).

Moreover, we found that the acetogenic Wood–Ljungdahl pathway (from carbon dioxide to acetate, including the tetrahydrofolate interconversion steps) was entirely covered by Firmicutes members, mainly Clostridiales. This pathway is used by acetogens to convert hydrogen and carbon dioxide into acetic acid, and its key importance is related to the oxidation of the hydrogen generated during the fermentation of dietary macromolecules (Koropatkin *et al*., 2012). In this study, we were able to identify all enzymatic players involved in this pathway, supporting its key relevance within microbial metabolism in sheep colon. More specifically, most enzymes involved in the tetrahydrofolate interconversion steps were taxonomically assigned not lower than the phylum level (always Firmicutes), indicating a high level of conservation within the Firmicutes members of the corresponding orthologous enzymes identified. Conversely, the key players of the last two reactions (from acetyl‐CoA to acetate, sequentially catalysed by phosphate acetyltransferase and acetate kinase) could be identified as being members of Clostridiales (including Lachnospiraceae and Oscillospiraceae), although a few peptides belonged to *Bacteroides* and *Prevotella* species from Bacteroidetes.

Furthermore, Bacteroidales were found to be responsible for most steps of the tricarboxylic acid cycle, as highlighted by a recent work on the effects of diet‐induced obesity on the mouse gut microbiota (Denou *et al*., 2016), whereas the enzyme RuBisCo, as expected, was detected only as expressed by photosynthetic Cyanobacteria. In addition, one key enzyme in the galactose metabolism, galactokinase, was found associated with Bacteroidetes members only. This enzyme was recently demonstrated as essential for a *Bacteroides* species to accomplish early colonization of the colonic microbiota (Yaung *et al*., 2015).

Lastly, it is worth noting that the phylum‐based distribution of the expressed carbon metabolism enzymes was different from the distribution of the same genes in the metagenome (Fig. S1), in line with the above‐stated observations.

### Focus on glycan import and degradation: ABC transporters, starch utilization system and glycosyl hydrolases

We then examined functions responsible for import and degradation of glycans, in view of the high content in glycans in the plant‐based sheep diet, and to evaluate the (residual) relevance of such activities in the sheep colon after the massive digestion of plant material performed in the rumen.

First, we focused on genes and proteins classified as ABC transporters, especially those capable of transporting mono‐ and oligosaccharides, which are listed in Table 2. These comprise both generic multiple sugar transport systems, such as msmX (Ferreira and Sa‐Nogueira, 2010), and more specific transporters, targeting mono‐ and disaccharides such as ribose and maltose (Oldham and Chen, 2011; Clifton *et al*., 2015). In most cases, according to both S‐MG and MP data, these functions were related to Firmicutes members (many different genera belonging to the order Clostridiales), followed by Actinobacteria (mainly Actinomycetales) for S‐MG and Spirochaetes (especially *Treponema*) for MP.

**Table 2:** Carbohydrate ABC transporter genes and proteins identified in the faecal microbiota of all sheep by metagenomics and metaproteomics respectively

Transported molecules	Identified gene(s)	Associated phyla	Identified protein(s)	Associated phyla
Aldouronate	*lplB, lplC*	Firmicutes	lplA	Firmicutes
Alpha glucoside	*aglK*		aglK	Spirochaetes
Arabinogalactan	*ganQ*	Firmicutes		
Arabinosaccharide	*araQ*	Firmicutes		
D‐Allose	*alsA*			
D‐Xylose	*xylG*			
L‐Arabinose	*araG*			
Maltose/maltodextrin	*malK*		malK	Firmicutes
Methyl‐galactoside	*mglA*	Firmicutes	mglB	Firmicutes
Multiple mono‐ and oligosaccharides	*msmX*	Firmicutes, Actinobacteria	msmX	Firmicutes
Multiple oligosaccharides	*gguA, gguS*	Firmicutes, Actinobacteria	gguS	Firmicutes
Myoinositol	*iatA*	Firmicutes		
Rhamnose	*rhaT*	Firmicutes		
Ribose/D‐xylose	*rbsA, rbsC*	Firmicutes, Actinobacteria	rbsB	Firmicutes
sn‐Glycerol‐3‐phosphate	*ugpC*	Firmicutes, Actinobacteria	ugpC	Firmicutes

As mentioned above, a large amount of peptides were assigned to the Bacteroidetes‐specific TonB‐dependent receptor family, namely to the starch utilization system (Sus) protein C, essential for complex carbohydrate degradation in Bacteroidetes (Reeves *et al*., 1997; Martens *et al*., 2009). The Sus proteins are located in the periplasm and the outer membrane and have the role of sequentially binding starch to the cell surface, degrading it into oligosaccharides and finally transporting the degradation products into the periplasmic space, where they are further digested into simpler sugars (e.g. glucose) and imported into the bacterial cell (Koropatkin *et al*., 2012). Of note, the percentage of identified peptides classified as belonging to the Ton‐B‐dependent receptor family was 20‐fold higher than the percentage of the corresponding genes sequenced, indicating a likely strong expression rate for this gene family.

Finally, we focused our attention on glycosyl hydrolases (GHs), because of their key role in degradation of plant biomass. We detected genes belonging to 56 different GH families in S‐MG, of which 28 were found in all animals (listed in Table S4). The most represented family was GH 13, mainly composed by alpha amylases, with 70% of genes belonging to Firmicutes; then, we found GH 3 (mainly beta‐glucosidases, 57% from Firmicutes and 41% from Bacteroidetes), GH 2 (mainly beta‐galactosidases, assigned at 51% to Firmicutes and 44% to Bacteroidetes) and GH 51 (64% Firmicutes and 31% Bacteroidetes). On the other hand, three GH families were found as expressed by the microbiota of all animals, according to MP data, namely GH 101 (clostridial endo‐alpha‐*N*‐acetylgalactosaminidase), GH 13 (pullulanase from Lachnospiraceae) and GH 94 (cellobiose phosphorylase, assigned to various phyla). Interestingly, the relative abundance of GH peptides identified was about sixfold lower than that of the corresponding genes sequenced, indicating a likely poor expression of this functional gene family, probably because of the relatively low amount of complex (and still undigested) polysaccharides that reach the colon after the extensive degradation occurred within the upper tracts of the ruminant digestive system (Huntington *et al*., 2006).

### Non‐microbial components detected in the faecal material through metaproteomics

The mass spectra generated in this work were also searched against a ‘generic’ database (without taxonomic filters towards microbial sequences) to achieve information about all organisms contained in the faecal samples. As a result, we found that besides spectra of microbial origin (about 30% of the total) approximately 45% of spectra were assigned to the host (phylum Chordata), while 13% could be attributed to plant material (with those assigned to Poaceae and Fabaceae families accounting for about 70% of them). Less than 1% of detected peptides were classified as belonging to further eukaryotic phyla as ‐ in decreasing abundance order ‒ Arthropoda, Mollusca, Nematoda, Annelida (including the *Hirudo* genus) and Platyhelminthes (including the *Fasciola* genus, comprising parasites of the small ruminant intestine). Even more interestingly, peptides from several protists known for intestinal tropism in sheep were also identified, including the following genera: *Entamoeba*,* Blastocystis*,* Andalucia*,* Giardia* and *Entodinium*, the last being a ciliate protozoan with a well‐known capability to digest and ferment starch, producing SCFAs (Belzecki *et al*., 2013).

In addition, based on S‐MG data, we were able to detect in four animals a few sequences attributed to *Haemonchus* (about 0.005% of annotated reads; an identified peptide sequence was also assigned to this genus). Of note, *Haemonchus contortus* is described as the most important gastroenteric nematode of sheep in many regions of the world, causing haemonchosis (Getachew *et al*., 2007).

## Conclusions

To the best of our knowledge, the results presented in this study represent the first ‘omic’ characterization of the sheep faecal microbiota to date. The application of a multi‐omic strategy, comprising V4‐MG, S‐MG and MP approaches, enabled us to take a comprehensive picture of both the taxonomic structure and the functional activity of the microbial communities inhabiting the distal tract of the ovine digestive system.

Under a taxonomic perspective, with Firmicutes being the main phylum, the sheep microbiota appeared globally comparable to that of other ruminants. In functional terms, we found that the sheep faecal microbiota was primarily involved in catabolism, and described several activities responsible for transport and degradation of carbohydrates. We also identified several phylum‐specific pathways, such as methanogenesis for Euryarchaeota and acetogenesis for Firmicutes. Furthermore, our approach provided information regarding the eukaryotic part of the microbiota (e.g. fungi and protists).

These findings, and the whole data set provided here, can be useful for deepening our understanding on organization and role of the large intestine microbiota in small ruminants, and pave the way to further studies investigating the relationship between gut microbiota dynamics and management and dietary variables key for sheep farming.

## Experimental procedures

### Animal description and faecal sample collection

Faecal samples were collected from the rectal ampulla of five lactating Sarda sheep in November 2012. Contact with the surrounding environment was minimized by transferring the faecal material immediately from the rectum to the collection tube. Sheep came from the same flock, were free‐grazing and fed a limited amount of commercial feed only during milking (max. 400 g per day) and were apparently healthy. All samples were immediately stored at −80°C until use. At the time of the analyses, samples were thawed at 4°C, and from each of them two stool fragments were collected for DNA and protein extraction respectively.

### DNA sample preparation

Stool samples were subjected to DL or DC as described earlier (Apajalahti *et al*., 1998; Tanca *et al*., 2015). Briefly, faecal samples (weighing approximately 100 mg each) were resuspended in PBS and subjected to low‐speed centrifugation to eliminate gross particulate material for a total of three rounds. The supernatants were then centrifuged at 20 000 *g* for 15 min, and the derivative pellets were subjected to DNA extraction. DNA extraction was performed in parallel using two commercial kits, namely the QIAamp Fast DNA Stool Kit (Qiagen, Hilden, Germany) and the E.Z.N.A. Soil DNA Kit (Omega Bio‐Tek, Norcross, GA, USA), according to the respective manufacturers’ instructions. DC‐pretreated samples were additionally subjected to DNA extraction according to the standard phenol/chloroform/isoamyl alcohol (25:24:1) method.

### 16S rDNA analysis

Primer design for universal amplification of the V4 region of 16S rDNA was based on a protocol published by Caporaso and co‐workers (Caporaso *et al*., 2011). PCR cycling conditions were as follows: 2 min at 94°C; 28 cycles of 30 s at 94°C, 30 s at 55°C, 2 min at 68°C; finally, 7 min at 72°C. PCR products were confirmed on 2% agarose gel (Sigma Aldrich, St Louis, MO, USA). Two separate 16S rRNA gene amplification reactions were performed, pooled together, cleaned up using AMPure XP (Beckman Coulter, Brea, CA, USA) magnetic beads and quantified with the Qubit HS assay using the Qubit fluorometer 2.0 (Life Technologies, Grand Island, NY, USA).

Libraries were constructed according to the Nextera XT kit (Illumina, San Diego, CA, USA). Sequence‐ready libraries were normalized to ensure equal library representation in the pooled samples. DNA sequencing was performed with the Illumina HiScanSQ sequencer, using the paired‐end method and 93 cycles of sequencing.

The Illumina demultiplexed paired reads were trimmed for the first 20 bp using FASTX, and the sequences with Nextera adapter contamination were identified using the UniVec database (ftp://ftp.ncbi.nlm.nih.gov/pub/UniVec/) and removed. Therefore, the paired reads with a minimum overlap of eight bases were merged using a specific QIIME script. OTU generation was performed using a QIIME pipeline based on USEARCH's OTU clustering recommendations (http://www.drive5.com/usearch/manual/otu_clustering.html). Reads were clustered at 97% identity using UCLUST to produce OTUs (Edgar, 2010). Taxonomy assignment of resulting OTUs was performed using the Greengenes 13_8 database (DeSantis *et al*., 2006). With taxonomic lineages in hand, OTU tables were computed using the QIIME 1.9.0 software suite (Caporaso *et al*., 2010; Kuczynski *et al*., 2010). The relative proportion of read counts was used as a quantitative estimation of the abundance of each taxon.

### Metagenome analysis

Libraries were constructed according to the Nextera XT kit and sequenced with the HiScanSQ sequencer (both from Illumina), using the paired‐end method and 93 cycles of sequencing.

Read processing was carried out using tools from the USEARCH suite v.8.1.1861 (Edgar, 2010; Edgar and Flyvbjerg, 2015), specifically merging of paired reads (fastq_mergepairs command, setting parameters as follows: fastq_truncqual 3, fastq_minovlen 8, fastq_maxdiffs 0) and quality filtering (fastq_filter command, with fastq_truncqual 15 and fastq_minlen 100).

Taxonomic annotation was performed using MEGAN v.5.10 (Huson and Mitra, 2012). Read sequences were preliminary subjected to DIAMOND (v.0.7.1) search against the NCBI‐nr DB (2014/05 update), using the blastx command with default parameters (Buchfink *et al*., 2015). Then, a lowest common ancestor classification was performed on DIAMOND results using MEGAN with default parameters.

Functional annotation was accomplished by DIAMOND blastx search (e‐value threshold 10^−5^) against bacterial sequences from the UniProt/Swiss‐Prot database (release 2014_12) and subsequent retrieval of protein family, KEGG orthologous group and pathway information associated with each UniProt/Swiss‐Prot accession number (UniProtConsortium, 2015).

The relative proportion of read counts was used as a quantitative estimation of the abundance of each taxon or function.

The metagenomic sequence data were deposited in the European Nucleotide Archive under the Project Accession Number PRJEB14312.

### Protein sample preparation

Stool samples (average weight 356 ± 31 mg) were resuspended by vortexing in SDS‐based extraction buffer and then heated and subjected to a combination of bead‐beating and freeze–thawing steps as detailed elsewhere (Tanca *et al*., 2014).

Protein extracts were subjected to on‐filter reduction, alkylation and trypsin digestion according to the filter‐aided sample preparation protocol (Wisniewski *et al*., 2009), with slight modifications detailed elsewhere (Tanca *et al*., 2013).

### Metaproteome analysis

LC‐MS/MS analysis was carried out using an LTQ‐Orbitrap Velos mass spectrometer (Thermo Scientific, San Jose, CA, USA) interfaced with an UltiMate 3000 RSLCnano LC system (Thermo Scientific). The single‐run 1D LC peptide separation was performed as previously described (Tanca *et al*., 2014), loading 4 μg of peptide mixture per each sample and applying a 485 min separation gradient. The mass spectrometer was set up in a data‐dependent MS/MS mode, with higher‐energy collision dissociation as the fragmentation method, as detailed elsewhere (Tanca *et al*., 2013). An average of 69 435 ± 870 spectra were acquired per sample.

Peptide identification was performed using the Proteome Discoverer informatic platform (version 1.4; Thermo Scientific), with Sequest‐HT as search engine and Percolator for peptide validation (FDR < 1%). Search parameters were set as described previously (Tanca *et al*., 2014).

Parallel searches were performed using three different sequence databases. The first database was composed by the metagenomic sequences obtained in this study, both as raw reads and as assembled contigs (8 595 757 sequences). Paired reads were merged using the script join_paired_ends.py inside the QIIME package, v.1.9 (Caporaso *et al*., 2010) with a minimum overlap of 8 base pairs. The output sequences were filtered (with a fastq_truncqual option = 15) and clustered at 100% using USEARCH v. 5.2.236 (Edgar, 2010). Read assembly into contigs was carried out using Velvet v.1.2.10 (Zerbino and Birney, 2008), by setting 61 as k‐mer length, 200 as insert length and 300 as minimum contig length. Open reading frames were found from both reads and contigs using FragGeneScan v.1.19, with the training for Illumina sequencing reads with about 0.5% error rate (Rho *et al*., 2010).

The second database was a selection of all bacterial, archaeal, fungal and gut microbiota sequences (79 203 800 sequences in total) from the 2015_02 release of the UniProtKB database.

The metaproteomic data (regarding the microbial component of the faecal material) were obtained by merging results of searches against the two above‐mentioned databases.

A third database (specifically, the whole UniProtKB database, release 2014_12, 89 136 540 sequences) was finally employed to achieve information only concerning the non‐microbial components of the sheep microbiota.

The relative proportion of spectral counts (peptide‐spectrum matches) was used as a quantitative estimation of the abundance of each taxon or function.

The mass spectrometry proteomics data have been deposited to the ProteomeXchange Consortium via the PRIDE (Vizcaino *et al*., 2016) partner repository with the data set identifier PXD004524.

Taxonomic and functional assignments were performed as described above for metagenome sequences, except using the DIAMOND blastp command instead of blastx.

## Conflict of Interest

None of the authors has any potential conflict of interest to declare.

## Supporting information


**Fig. S1.** Enzymatic functions identified by shotgun metagenomics and mapped in the KEGG carbon metabolism pathway. Coloured arrows indicate enzymes detected in all animals, with the colour corresponding to the main phylum to which the function was assigned (red, Bacteroidetes; blue, Firmicutes; brown, Actinobacteria). Grey arrows indicate enzymes detected in at least one but not all animals, or not assigned unambiguously to at least one phylum.

**Table S1.** DNA sequencing, peptide identification and taxonomic/functional annotation metrics.

**Table S2.** Protein families detected in all animals by metaproteomics.

**Table S3.** Gene families assigned to a unique phylum and detected in all animals by shotgun metagenomics.

**Table S4.** Glycosyl hydrolase families detected in all animals by shotgun metagenomics.

**Data S1.** 16S/V4 rDNA sequencing data, aggregated at different levels (OTU, phylum, class, order, family, genus).

**Data S2.** Shotgun metagenomics data, aggregated at different taxonomic levels (phylum, class, order, family, genus), according to different functional categories (KOG, gene family, pathway) and based on a combined functional–taxonomic classification (gene family + phylum, pathway + phylum).

**Data S3.** Metaproteomics data, aggregated at different taxonomic levels (phylum, class, order, family, genus), according to different functional categories (KOG, protein family, pathway) and based on a combined functional–taxonomic classification (protein family + phylum, pathway + phylum).
