# Supplementary figures and images for: Diversity and functions of the sheep faecal microbiota: a multi‐omic characterization
Source: Microb Biotechnol. 2017 Feb 6;10(3):541–54. doi: 10.1111/1751-7915.12462 (PMC5404191; doi:10.1111/1751-7915.12462)

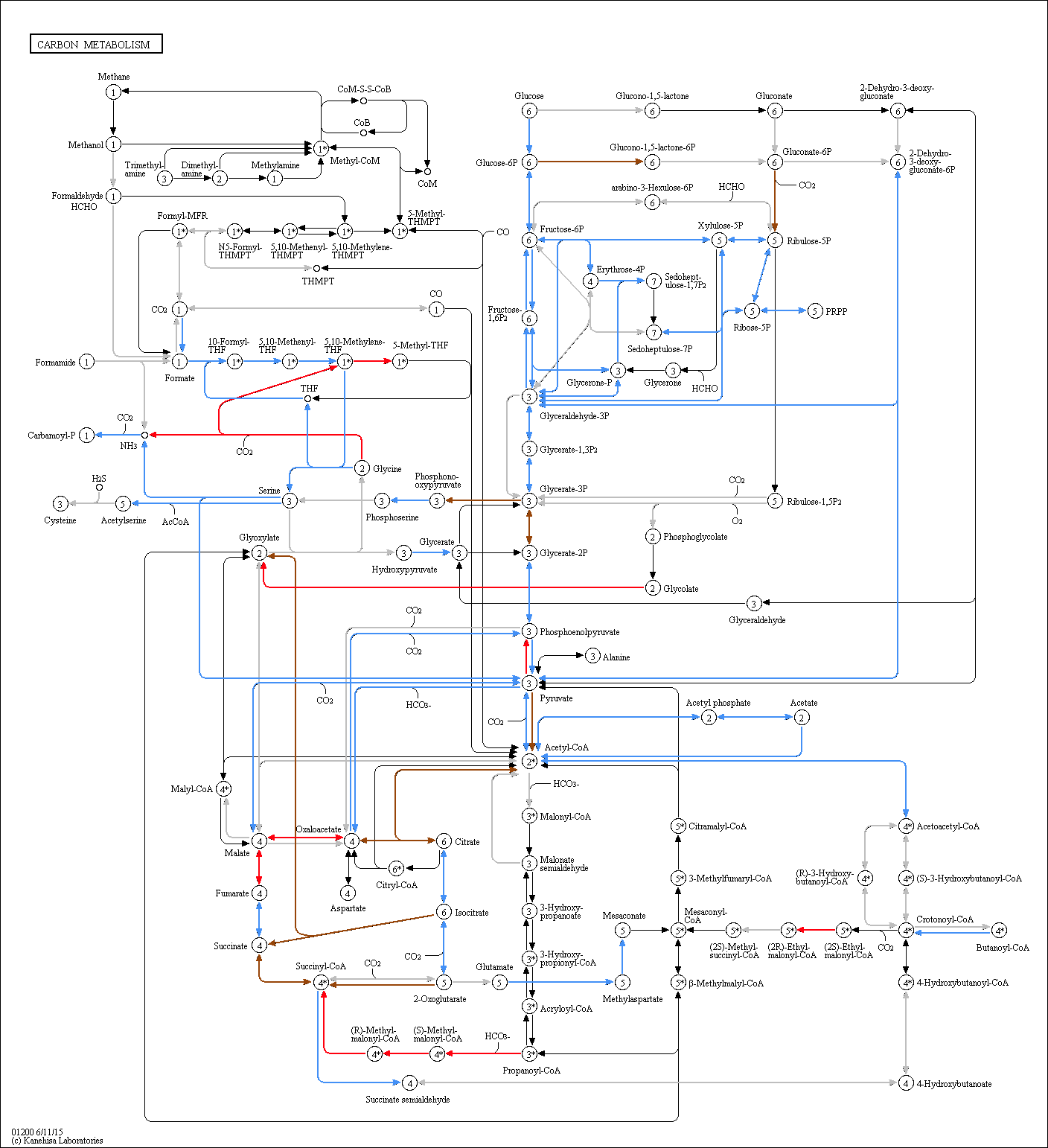

Supplement: Supplementary file 1 — Fig. S1. Enzymatic functions identified by shotgun metagenomics and mapped in the KEGG carbon metabolism pathway. Coloured arrows indicate enzymes detected in all animals, with the colour corresponding to the main phylum to which the function was assigned (red, Bacteroidetes; blue, Firmicutes; brown, Actinobacteria). Grey arrows indicate enzymes detected in at least one but not all animals, or not assigned unambiguously to at least one phylum. [file MBT2-10-541-s001.png]
