# Supplementary material for: Diversity and functions of the sheep faecal microbiota: a multi‐omic characterization
Source: Microb Biotechnol. 2017 Feb 6;10(3):541–54. doi: 10.1111/1751-7915.12462 (PMC5404191; doi:10.1111/1751-7915.12462)
Supplement: Supplementary file 2 — Table S1. DNA sequencing, peptide identification and taxonomic/functional annotation metrics. [file MBT2-10-541-s002.docx]

**Table S1.** DNA sequencing, peptide identification and taxonomic/functional annotation metrics.

|  | **16S/V4 rDNA analysis** | | **Shotgun metagenomic analysis** | | | **Metaproteomic analysis** | | | | |
| --- | --- | --- | --- | --- | --- | --- | --- | --- | --- | --- |
| **Sample** | **reads obtained** | **taxonomically annotated reads (phylum)** | **reads obtained** | **taxonomically annotated reads (phylum)** | **functionally annotated reads^§^** | **spectra obtained** | **unique peptides identified^#^** | **peptide-spectrum matches (PSMs)^#^** | **taxonomically annotated PSMs (phylum)** | **functionally annotated PSMs^§^** |
| **1** | 715,296 | 317,119 | 68,423 | 24,901 | 10,387 | 69,646 | 3,138 | 7,679 | 2,735 | 3,615 |
| **2** | 721,181 | 316,778 | 4,164,588 | 1,724,300 | 620,979 | 69,772 | 5,890 | 14,672 | 5,309 | 7,021 |
| **3** | 549,000 | 247,133 | 1,083,477 | 426,805 | 168,785 | 70,223 | 4,350 | 10,729 | 3,755 | 5,163 |
| **4** | 337,529 | 150,311 | 1,074,150 | 402,746 | 166,702 | 69,593 | 4,420 | 10,864 | 3,869 | 5,227 |
| **5** | 733,641 | 335,452 | 2,135,821 | 826,163 | 319,957 | 67,943 | 3,871 | 10,327 | 3,464 | 4,661 |

^#^ Peptide identifications were obtained searching in parallel against two sequence databases (DB1, metagenomic sequences obtained in this study, both as raw reads and assembled contigs; DB2: a selection of all bacterial, archaeal, fungal and gut microbiota sequences from the 2015_02 release of the UniProtKB database).

**^§^** A read/peptide sequence was considered as “functional annotated” when it was assigned a specific UniProt/Swiss-Prot accession number upon blast alignment.
