# Supplementary material for: Diversity and functions of the sheep faecal microbiota: a multi‐omic characterization
Source: Microb Biotechnol. 2017 Feb 6;10(3):541–54. doi: 10.1111/1751-7915.12462 (PMC5404191; doi:10.1111/1751-7915.12462)
Supplement: Supplementary file 3 — Table S2. Protein families detected in all animals by metaproteomics. [file MBT2-10-541-s003.docx]

**Table S2.** Protein families detected in all animals by metaproteomics.

| **Protein family** | **Sheep 1** | **Sheep 2** | **Sheep 3** | **Sheep 4** | **Sheep 5** |
| --- | --- | --- | --- | --- | --- |
| [NiFe]/[NiFeSe] hydrogenase large subunit | 0.29860% | 0.04566% | 0.33099% | 0.26738% | 0.62183% |
| 3-hydroxyacyl-CoA dehydrogenase | 0.05972% | 0.10653% | 0.06206% | 0.02057% | 0.11515% |
| AAA ATPase | 0.05972% | 0.06087% | 0.06206% | 0.06170% | 0.13819% |
| ABC transporter | 2.56793% | 1.01963% | 1.55151% | 2.03620% | 1.24367% |
| AccD/PCCB | 0.47775% | 0.35002% | 0.31030% | 0.43192% | 0.36849% |
| Acetokinase | 0.20902% | 0.47177% | 0.51717% | 0.43192% | 0.20728% |
| Acetyl-CoA hydrolase/transferase | 0.20902% | 0.22828% | 0.22755% | 0.18511% | 0.11515% |
| Actin | 1.79158% | 1.35444% | 1.84113% | 1.50144% | 1.88853% |
| Acyl-CoA dehydrogenase | 1.28397% | 0.48699% | 0.78610% | 0.51419% | 0.36849% |
| Acyl-CoA mutase large subunit | 0.05972% | 0.06087% | 0.06206% | 0.06170% | 0.13819% |
| AdoMet synthase | 0.02986% | 0.07609% | 0.06206% | 0.06170% | 0.06909% |
| AhpC/TSA | 0.11944% | 0.10653% | 0.06206% | 0.14397% | 0.09212% |
| AIR synthase | 0.02986% | 0.18262% | 0.02069% | 0.22624% | 0.06909% |
| Aldolase class II | 0.02986% | 0.07609% | 0.06206% | 0.02057% | 0.02303% |
| Archaeal histone HMF | 0.05972% | 0.06087% | 0.02069% | 0.04114% | 0.04606% |
| ATCase/OTCase | 0.08958% | 0.10653% | 0.16549% | 0.04114% | 0.18425% |
| ATPase alpha/beta chains | 1.52284% | 1.53706% | 1.65494% | 1.54258% | 2.18793% |
| ATPase C chain | 1.16453% | 0.54786% | 0.82747% | 1.00782% | 1.08245% |
| Bacterial flagellin | 1.55270% | 1.40009% | 1.59288% | 1.27520% | 2.21096% |
| Bacterial histone-like protein | 0.14930% | 0.25871% | 0.06206% | 0.34965% | 0.20728% |
| Bacterial microcompartments protein | 0.83607% | 0.66961% | 0.70335% | 0.57589% | 0.69093% |
| Bacterial solute-binding protein 1 | 0.11944% | 0.07609% | 0.08275% | 0.04114% | 0.09212% |
| Bacterial solute-binding protein 2 | 1.25411% | 0.50221% | 0.68266% | 0.84327% | 1.03639% |
| Bacterial solute-binding protein 7 | 0.02986% | 0.01522% | 0.06206% | 0.02057% | 0.02303% |
| Bacterial/plant glucose-1-phosphate adenylyltransferase | 0.17916% | 0.39568% | 0.45511% | 0.14397% | 0.04606% |
| Band 7/mec-2 | 0.14930% | 0.09131% | 0.14481% | 0.06170% | 0.11515% |
| Beta-eliminating lyase | 0.08958% | 0.22828% | 0.20687% | 0.30852% | 0.11515% |
| CdhC | 0.08958% | 0.16740% | 0.10343% | 0.08227% | 0.04606% |
| Chaperonin (HSP60) | 0.38818% | 1.17182% | 0.74472% | 0.80214% | 1.49701% |
| Citrate synthase | 0.02986% | 0.01522% | 0.02069% | 0.02057% | 0.02303% |
| Class I fructose-bisphosphate aldolase | 0.05972% | 0.04566% | 0.10343% | 0.02057% | 0.04606% |
| Class II fructose-bisphosphate aldolase | 1.61242% | 2.17623% | 2.19280% | 2.20074% | 2.39521% |
| Class-I fumarase | 0.08958% | 0.06087% | 0.02069% | 0.04114% | 0.04606% |
| Class-I pyridine nucleotide-disulfide oxidoreductase | 0.17916% | 0.07609% | 0.14481% | 0.08227% | 0.13819% |
| Class-I pyridoxal-phosphate-dependent aminotransferase | 0.32846% | 0.09131% | 0.02069% | 0.18511% | 0.06909% |
| Class-II aminoacyl-tRNA synthetase | 0.50761% | 0.56308% | 0.43442% | 0.59646% | 0.43759% |
| Class-III pyridoxal-phosphate-dependent aminotransferase | 0.35832% | 0.10653% | 0.31030% | 0.20568% | 0.34546% |
| Class-V pyridoxal-phosphate-dependent aminotransferase | 0.74649% | 0.89788% | 0.59992% | 0.82271% | 0.59880% |
| ClpA/ClpB | 0.11944% | 0.21306% | 0.04137% | 0.10284% | 0.23031% |
| Complex I 51 kDa subunit | 0.47775% | 0.56308% | 0.57923% | 0.41135% | 0.34546% |
| Diol/glycerol dehydratase large subunit | 0.14930% | 0.03044% | 0.06206% | 0.06170% | 0.02303% |
| Diol/glycerol dehydratase medium subunit | 0.08958% | 0.07609% | 0.06206% | 0.12341% | 0.09212% |
| Diol/glycerol dehydratase small subunit | 0.23888% | 0.13697% | 0.08275% | 0.26738% | 0.16122% |
| D-isomer specific 2-hydroxyacid dehydrogenase | 0.14930% | 0.04566% | 0.14481% | 0.16454% | 0.04606% |
| DsrC/TusE | 0.05972% | 0.01522% | 0.04137% | 0.06170% | 0.06909% |
| EF-Ts | 0.17916% | 0.44133% | 0.31030% | 0.30852% | 0.11515% |
| Elongation factor P | 0.05972% | 0.19784% | 0.14481% | 0.18511% | 0.25334% |
| Enolase | 0.23888% | 0.21306% | 0.28962% | 0.37022% | 0.80608% |
| Enoyl-CoA hydratase/isomerase | 0.02986% | 0.04566% | 0.02069% | 0.02057% | 0.04606% |
| ETF alpha-subunit/FixB | 0.26874% | 0.27393% | 0.24824% | 0.34965% | 0.16122% |
| ETF beta-subunit/FixA | 0.98537% | 0.54786% | 0.55854% | 0.51419% | 0.23031% |
| Eukaryotic mitochondrial porin | 0.02986% | 0.01522% | 0.04137% | 0.02057% | 0.02303% |
| EutC | 0.05972% | 0.01522% | 0.06206% | 0.08227% | 0.13819% |
| EutL/PduB | 0.32846% | 0.10653% | 0.12412% | 0.16454% | 0.09212% |
| ExbB/TolQ | 0.08958% | 0.03044% | 0.08275% | 0.02057% | 0.06909% |
| FAD-dependent oxidoreductase 2 | 0.98537% | 0.65439% | 0.68266% | 0.49362% | 0.80608% |
| FGGY kinase | 0.56733% | 0.30437% | 0.41374% | 0.51419% | 0.36849% |
| FldB/FldC dehydratase beta subunit | 0.44789% | 0.16740% | 0.16549% | 0.22624% | 0.02303% |
| Formate--tetrahydrofolate ligase | 1.31383% | 0.85223% | 0.93091% | 0.69930% | 0.71396% |
| FrhB | 0.08958% | 0.01522% | 0.06206% | 0.08227% | 0.11515% |
| Gfo/Idh/MocA | 0.02986% | 0.03044% | 0.02069% | 0.02057% | 0.06909% |
| GHMP kinase | 0.02986% | 0.09131% | 0.06206% | 0.04114% | 0.09212% |
| Glu/Leu/Phe/Val dehydrogenases | 4.89698% | 3.10455% | 3.35126% | 2.77664% | 4.00737% |
| Glucosamine/galactosamine-6-phosphate isomerase | 0.23888% | 0.31959% | 0.35168% | 0.24681% | 0.13819% |
| Glutamine synthetase | 0.02986% | 0.04566% | 0.04137% | 0.04114% | 0.06909% |
| Glyceraldehyde-3-phosphate dehydrogenase | 5.43446% | 5.79820% | 6.24741% | 6.39654% | 6.77107% |
| Glycogen phosphorylase | 0.05972% | 0.04566% | 0.04137% | 0.08227% | 0.06909% |
| Glycosyl hydrolase 101 | 0.08958% | 0.10653% | 0.22755% | 0.10284% | 0.02303% |
| Glycosyl hydrolase 13 | 0.14930% | 0.03044% | 0.14481% | 0.08227% | 0.11515% |
| Glycosyl hydrolase 94 | 0.05972% | 0.10653% | 0.04137% | 0.06170% | 0.06909% |
| Glycosyltransferase 1 | 0.11944% | 0.28915% | 0.39305% | 0.22624% | 0.11515% |
| Glyoxalase I | 0.08958% | 0.04566% | 0.08275% | 0.08227% | 0.04606% |
| GPI | 0.32846% | 0.31959% | 0.28962% | 0.10284% | 0.20728% |
| Group II decarboxylase | 0.17916% | 0.28915% | 0.26893% | 0.14397% | 0.18425% |
| GSP E | 0.02986% | 0.03044% | 0.04137% | 0.04114% | 0.04606% |
| H(+)-translocating pyrophosphatase (TC 3.A.10) | 0.11944% | 0.07609% | 0.08275% | 0.08227% | 0.25334% |
| Heat shock protein 70 | 0.77635% | 1.82621% | 1.42739% | 1.27520% | 1.42791% |
| Heat shock protein 90 | 0.68677% | 0.44133% | 0.55854% | 0.47306% | 0.50668% |
| Hfq | 0.02986% | 0.01522% | 0.02069% | 0.02057% | 0.02303% |
| Histone H2B | 0.11944% | 0.01522% | 0.04137% | 0.04114% | 0.06909% |
| Histone H4 | 0.50761% | 0.33480% | 0.49648% | 0.34965% | 0.41456% |
| IMPDH/GMPR | 0.11944% | 0.15218% | 0.26893% | 0.10284% | 0.20728% |
| Iron-containing alcohol dehydrogenase | 0.17916% | 0.10653% | 0.35168% | 0.24681% | 0.20728% |
| Isocitrate and isopropylmalate dehydrogenases | 0.17916% | 0.13697% | 0.22755% | 0.16454% | 0.11515% |
| Ketol-acid reductoisomerase | 0.14930% | 0.22828% | 0.16549% | 0.20568% | 0.13819% |
| KHG/KDPG aldolase | 0.26874% | 0.19784% | 0.22755% | 0.04114% | 0.04606% |
| LDH/MDH | 0.65691% | 0.33480% | 0.49648% | 0.45249% | 0.66789% |
| L-fucose isomerase | 0.53747% | 0.45655% | 0.51717% | 0.39079% | 0.55274% |
| Mer | 0.65691% | 0.33480% | 0.68266% | 0.65817% | 0.80608% |
| Methylaspartate ammonia-lyase | 0.02986% | 0.07609% | 0.16549% | 0.10284% | 0.18425% |
| Methylmalonyl-CoA mutase | 0.53747% | 0.53264% | 0.55854% | 1.17236% | 0.92123% |
| MIP/aquaporin (TC 1.A.8) | 0.05972% | 0.01522% | 0.02069% | 0.04114% | 0.04606% |
| Mitochondrial carrier (TC 2.A.29) | 0.02986% | 0.03044% | 0.02069% | 0.04114% | 0.04606% |
| MTD | 0.08958% | 0.04566% | 0.06206% | 0.12341% | 0.16122% |
| MtrA | 0.08958% | 0.04566% | 0.12412% | 0.08227% | 0.13819% |
| Myoviridae tail sheath protein | 0.20902% | 0.10653% | 0.18618% | 0.16454% | 0.11515% |
| NAD(P)-dependent epimerase/dehydratase | 0.29860% | 0.30437% | 0.24824% | 0.20568% | 0.11515% |
| NADH:flavin oxidoreductase/NADH oxidase | 0.02986% | 0.01522% | 0.02069% | 0.02057% | 0.04606% |
| NagA | 0.05972% | 0.09131% | 0.16549% | 0.10284% | 0.04606% |
| NDK | 0.38818% | 0.15218% | 0.16549% | 0.20568% | 0.18425% |
| Ni-containing carbon monoxide dehydrogenase | 0.41804% | 0.53264% | 0.53786% | 0.39079% | 0.18425% |
| NifJ | 2.47835% | 1.79577% | 2.19280% | 2.38585% | 1.54307% |
| N-Me-Phe pilin | 0.02986% | 0.01522% | 0.02069% | 0.06170% | 0.04606% |
| OmpA | 0.23888% | 0.30437% | 0.16549% | 0.59646% | 0.39152% |
| PAL/histidase | 0.17916% | 0.03044% | 0.10343% | 0.02057% | 0.04606% |
| Peptidase S41A | 0.02986% | 0.01522% | 0.02069% | 0.04114% | 0.02303% |
| PEP-utilizing enzyme | 3.46372% | 4.50464% | 4.28217% | 4.29864% | 4.51405% |
| Phosphate acetyltransferase and butyryltransferase | 0.35832% | 0.45655% | 0.41374% | 0.37022% | 0.32243% |
| Phosphoenolpyruvate carboxykinase [ATP] | 0.98537% | 1.49140% | 1.15846% | 1.23406% | 1.01336% |
| Phosphoenolpyruvate carboxykinase [GTP] | 0.17916% | 0.60874% | 0.22755% | 0.53476% | 0.36849% |
| Phosphofructokinase type A (PFKA) | 0.44789% | 0.79136% | 0.49648% | 0.55533% | 0.43759% |
| Phosphoglycerate kinase | 1.28397% | 1.85664% | 1.59288% | 1.93336% | 1.35882% |
| Phosphohexose mutase | 0.11944% | 0.18262% | 0.16549% | 0.24681% | 0.13819% |
| PNP/UDP phosphorylase | 0.05972% | 0.06087% | 0.08275% | 0.04114% | 0.04606% |
| Polyribonucleotide nucleotidyltransferase | 0.11944% | 0.39568% | 0.10343% | 0.18511% | 0.23031% |
| PsaA/PsaB | 0.35832% | 0.15218% | 0.22755% | 0.22624% | 0.34546% |
| PsaD | 0.02986% | 0.01522% | 0.02069% | 0.04114% | 0.02303% |
| PsbB/PsbC | 0.05972% | 0.03044% | 0.04137% | 0.04114% | 0.04606% |
| PurH | 0.08958% | 0.09131% | 0.06206% | 0.10284% | 0.09212% |
| Purine/pyrimidine phosphoribosyltransferase | 0.02986% | 0.07609% | 0.04137% | 0.08227% | 0.04606% |
| Radical SAM | 0.05972% | 0.03044% | 0.06206% | 0.06170% | 0.06909% |
| Reaction center PufL/M/PsbA/D | 0.56733% | 0.30437% | 0.39305% | 0.63760% | 0.27637% |
| RecA | 0.02986% | 0.19784% | 0.20687% | 0.08227% | 0.16122% |
| Resistance-nodulation-cell division (RND) (TC 2.A.6) | 0.02986% | 0.04566% | 0.02069% | 0.02057% | 0.02303% |
| Ribose-phosphate pyrophosphokinase | 0.11944% | 0.06087% | 0.06206% | 0.12341% | 0.04606% |
| Ribosomal protein L10P | 0.29860% | 0.38046% | 0.43442% | 0.28795% | 0.23031% |
| Ribosomal protein L11P | 0.62705% | 0.98919% | 0.57923% | 0.69930% | 0.55274% |
| Ribosomal protein L12P | 0.02986% | 0.01522% | 0.04137% | 0.04114% | 0.06909% |
| Ribosomal protein L13P | 0.38818% | 0.47177% | 0.49648% | 0.28795% | 0.20728% |
| Ribosomal protein L14P | 0.77635% | 0.51743% | 0.53786% | 0.53476% | 0.50668% |
| Ribosomal protein L15P | 0.20902% | 0.45655% | 0.35168% | 0.39079% | 0.25334% |
| Ribosomal protein L16P | 0.86593% | 0.70005% | 0.64129% | 0.67873% | 0.39152% |
| Ribosomal protein L17P | 0.17916% | 0.22828% | 0.14481% | 0.10284% | 0.23031% |
| Ribosomal protein L18P | 0.23888% | 0.25871% | 0.16549% | 0.28795% | 0.11515% |
| Ribosomal protein L19P | 0.17916% | 0.31959% | 0.43442% | 0.12341% | 0.13819% |
| Ribosomal protein L1P | 0.86593% | 0.85223% | 1.01365% | 0.57589% | 0.62183% |
| Ribosomal protein L20P | 0.14930% | 0.16740% | 0.10343% | 0.02057% | 0.04606% |
| Ribosomal protein L21P | 0.08958% | 0.31959% | 0.20687% | 0.12341% | 0.06909% |
| Ribosomal protein L22P | 0.14930% | 0.09131% | 0.31030% | 0.08227% | 0.09212% |
| Ribosomal protein L23P | 0.29860% | 0.28915% | 0.31030% | 0.26738% | 0.13819% |
| Ribosomal protein L29P | 0.44789% | 0.30437% | 0.18618% | 0.34965% | 0.27637% |
| Ribosomal protein L2P | 0.17916% | 0.51743% | 0.39305% | 0.41135% | 0.16122% |
| Ribosomal protein L30P | 0.11944% | 0.22828% | 0.16549% | 0.08227% | 0.13819% |
| Ribosomal protein L31P | 0.02986% | 0.10653% | 0.12412% | 0.10284% | 0.06909% |
| Ribosomal protein L3P | 0.08958% | 0.48699% | 0.18618% | 0.20568% | 0.29940% |
| Ribosomal protein L4P | 0.11944% | 0.22828% | 0.20687% | 0.16454% | 0.04606% |
| Ribosomal protein L5P | 2.53807% | 1.62837% | 1.73769% | 1.76882% | 1.91156% |
| Ribosomal protein L6P | 0.23888% | 0.47177% | 0.43442% | 0.26738% | 0.20728% |
| Ribosomal protein L7/L12P | 0.83607% | 1.85664% | 1.28258% | 1.70712% | 2.02672% |
| Ribosomal protein S10P | 0.56733% | 0.44133% | 0.35168% | 0.30852% | 0.55274% |
| Ribosomal protein S11P | 0.17916% | 0.36524% | 0.59992% | 0.34965% | 0.32243% |
| Ribosomal protein S12P | 0.17916% | 0.35002% | 0.20687% | 0.45249% | 0.16122% |
| Ribosomal protein S13P | 0.68677% | 0.57830% | 0.59992% | 0.51419% | 0.55274% |
| Ribosomal protein S15P | 0.11944% | 0.27393% | 0.08275% | 0.26738% | 0.11515% |
| Ribosomal protein S16P | 0.05972% | 0.22828% | 0.28962% | 0.12341% | 0.11515% |
| Ribosomal protein S17P | 0.14930% | 0.19784% | 0.10343% | 0.10284% | 0.06909% |
| Ribosomal protein S19P | 0.11944% | 0.39568% | 0.31030% | 0.30852% | 0.41456% |
| Ribosomal protein S1P | 0.14930% | 0.22828% | 0.10343% | 0.18511% | 0.13819% |
| Ribosomal protein S2P | 1.13467% | 0.98919% | 0.99297% | 1.02838% | 0.78305% |
| Ribosomal protein S3P | 1.37354% | 0.73048% | 0.72404% | 0.59646% | 0.41456% |
| Ribosomal protein S4P | 0.44789% | 0.71526% | 0.51717% | 0.53476% | 0.32243% |
| Ribosomal protein S5P | 1.07495% | 0.92832% | 0.72404% | 0.67873% | 0.43759% |
| Ribosomal protein S6P | 0.08958% | 0.06087% | 0.06206% | 0.06170% | 0.02303% |
| Ribosomal protein S7P | 0.80621% | 1.14138% | 1.01365% | 0.76100% | 0.87517% |
| Ribosomal protein S8P | 1.22425% | 0.92832% | 0.95159% | 0.71987% | 0.64486% |
| Ribosomal protein S9P | 0.38818% | 0.45655% | 0.39305% | 0.24681% | 0.29940% |
| RNA polymerase alpha chain | 0.20902% | 0.36524% | 0.28962% | 0.37022% | 0.20728% |
| RNA polymerase beta chain | 0.71663% | 0.66961% | 0.57923% | 0.67873% | 0.52971% |
| RNA polymerase beta' chain | 0.50761% | 0.54786% | 0.45511% | 0.45249% | 0.36849% |
| RRF | 0.20902% | 0.24349% | 0.24824% | 0.24681% | 0.23031% |
| RuBisCO activase | 0.35832% | 0.12175% | 0.16549% | 0.22624% | 0.13819% |
| RuBisCO large chain | 0.83607% | 0.24349% | 0.39305% | 0.55533% | 0.69093% |
| RuBisCO small chain | 0.05972% | 0.01522% | 0.06206% | 0.08227% | 0.16122% |
| SAICAR synthetase | 0.05972% | 0.18262% | 0.08275% | 0.10284% | 0.06909% |
| SHMT | 0.11944% | 0.21306% | 0.22755% | 0.20568% | 0.13819% |
| Short-chain dehydrogenases/reductases (SDR) | 0.68677% | 0.35002% | 0.47580% | 0.16454% | 0.23031% |
| Small GTPase | 0.29860% | 0.15218% | 0.20687% | 0.24681% | 0.23031% |
| SMC | 0.05972% | 0.04566% | 0.04137% | 0.02057% | 0.02303% |
| Succinate dehydrogenase/fumarate reductase iron-sulfur protein | 0.14930% | 0.15218% | 0.14481% | 0.10284% | 0.13819% |
| Tetrahydrofolate dehydrogenase/cyclohydrolase | 0.02986% | 0.03044% | 0.10343% | 0.04114% | 0.02303% |
| Thiolase | 0.26874% | 0.15218% | 0.10343% | 0.06170% | 0.02303% |
| TonB-dependent receptor | 1.10481% | 1.26313% | 1.17915% | 1.52201% | 2.00368% |
| TRAFAC class translation factor GTPase | 12.66050% | 15.26404% | 15.70128% | 17.58536% | 18.30953% |
| Transketolase | 0.02986% | 0.06087% | 0.04137% | 0.08227% | 0.04606% |
| Triosephosphate isomerase | 1.43326% | 1.24791% | 0.95159% | 1.11065% | 0.82911% |
| Ubiquitin | 0.26874% | 0.12175% | 0.26893% | 0.22624% | 0.16122% |
| UDP-glucose/GDP-mannose dehydrogenase | 0.02986% | 0.03044% | 0.10343% | 0.02057% | 0.06909% |
| V-ATPase proteolipid subunit | 0.08958% | 0.04566% | 0.08275% | 0.02057% | 0.04606% |
| Vitamin-B12 dependent methionine synthase | 0.14930% | 0.09131% | 0.16549% | 0.08227% | 0.11515% |
| Xanthine dehydrogenase | 1.70200% | 0.42611% | 0.76541% | 0.47306% | 0.48365% |
| Xylose isomerase | 0.05972% | 0.13697% | 0.10343% | 0.20568% | 0.04606% |
