# Supplementary material for: Diversity and functions of the sheep faecal microbiota: a multi‐omic characterization
Source: Microb Biotechnol. 2017 Feb 6;10(3):541–54. doi: 10.1111/1751-7915.12462 (PMC5404191; doi:10.1111/1751-7915.12462)
Supplement: Supplementary file 4 — Table S3. Gene families assigned to a unique phylum and detected in all animals by shotgun metagenomics. [file MBT2-10-541-s004.docx]

**Table S3.** Gene families assigned to a unique phylum and detected in all animals by shotgun metagenomics.

| **Gene family** | **Phylum** | **Sheep 1** | **Sheep 2** | **Sheep 3** | **Sheep 4** | **Sheep 5** | ***Mean*** |
| --- | --- | --- | --- | --- | --- | --- | --- |
| Pup ligase/Pup deamidase | Actinobacteria | 0.016% | 0.001% | 0.012% | 0.056% | 0.011% | ***0.019%*** |
| Acetamidase/formamidase | Actinobacteria | 0.032% | 0.001% | 0.004% | 0.032% | 0.004% | ***0.014%*** |
| Prokaryotic pantothenate kinase | Actinobacteria | 0.016% | 0.002% | 0.003% | 0.025% | 0.008% | ***0.011%*** |
| Class-II DAHP synthase | Actinobacteria | 0.016% | 0.002% | 0.005% | 0.025% | 0.006% | ***0.011%*** |
| NucS endonuclease | Actinobacteria | 0.016% | 0.001% | 0.005% | 0.023% | 0.008% | ***0.011%*** |
| CAMP phosphodiesterase class-III | Actinobacteria | 0.016% | 0.001% | 0.004% | 0.022% | 0.007% | ***0.010%*** |
| WhiB | Actinobacteria | 0.032% | 0.001% | 0.002% | 0.012% | 0.002% | ***0.010%*** |
| Uricase | Actinobacteria | 0.016% | 0.000% | 0.005% | 0.026% | 0.001% | ***0.010%*** |
| PrpD | Actinobacteria | 0.016% | 0.000% | 0.005% | 0.015% | 0.006% | ***0.008%*** |
| UPF0678 | Actinobacteria | 0.016% | 0.001% | 0.005% | 0.010% | 0.003% | ***0.007%*** |
| Bacterial non-heme bromo- and chloro-peroxidases | Actinobacteria | 0.016% | 0.000% | 0.003% | 0.008% | 0.004% | ***0.006%*** |
| Lsr2 | Actinobacteria | 0.016% | 0.000% | 0.001% | 0.005% | 0.002% | ***0.005%*** |
| Glycosyl hydrolase 97 | Bacteroidetes | 0.111% | 0.185% | 0.100% | 0.081% | 0.121% | ***0.119%*** |
| PduL | Firmicutes | 0.127% | 0.055% | 0.063% | 0.072% | 0.046% | ***0.072%*** |
| Anti-sigma-factor | Firmicutes | 0.032% | 0.041% | 0.057% | 0.036% | 0.060% | ***0.045%*** |
| UPF0348 | Firmicutes | 0.032% | 0.047% | 0.047% | 0.034% | 0.050% | ***0.042%*** |
| Peptidase A25 | Firmicutes | 0.047% | 0.032% | 0.053% | 0.030% | 0.038% | ***0.040%*** |
| NEMF | Firmicutes | 0.063% | 0.023% | 0.033% | 0.034% | 0.037% | ***0.038%*** |
| GerABKA | Firmicutes | 0.016% | 0.033% | 0.041% | 0.033% | 0.065% | ***0.037%*** |
| TelA | Firmicutes | 0.032% | 0.023% | 0.040% | 0.038% | 0.043% | ***0.035%*** |
| UPF0297 | Firmicutes | 0.032% | 0.029% | 0.037% | 0.046% | 0.022% | ***0.033%*** |
| SleB | Firmicutes | 0.047% | 0.017% | 0.025% | 0.015% | 0.029% | ***0.027%*** |
| UPF0735 | Firmicutes | 0.000% | 0.025% | 0.044% | 0.026% | 0.034% | ***0.026%*** |
| UPF0340 | Firmicutes | 0.016% | 0.023% | 0.027% | 0.021% | 0.027% | ***0.023%*** |
| Gluconeogenesis factor | Firmicutes | 0.016% | 0.019% | 0.032% | 0.008% | 0.030% | ***0.021%*** |
| KtrA potassium transport | Firmicutes | 0.032% | 0.011% | 0.018% | 0.015% | 0.027% | ***0.020%*** |
| UPF0122 | Firmicutes | 0.032% | 0.016% | 0.023% | 0.009% | 0.020% | ***0.020%*** |
| Spermidine/spermine synthase | Firmicutes | 0.032% | 0.014% | 0.022% | 0.007% | 0.021% | ***0.019%*** |
| EutH | Firmicutes | 0.032% | 0.014% | 0.009% | 0.024% | 0.011% | ***0.018%*** |
| CodY | Firmicutes | 0.016% | 0.008% | 0.014% | 0.006% | 0.026% | ***0.014%*** |
| CDP-glycerol glycerophosphotransferase | Firmicutes | 0.032% | 0.007% | 0.011% | 0.015% | 0.002% | ***0.013%*** |
| LytR/CpsA/Psr regulatory protein | Firmicutes | 0.000% | 0.008% | 0.017% | 0.003% | 0.033% | ***0.012%*** |
| FldB/FldC dehydratase beta subunit | Firmicutes | 0.016% | 0.008% | 0.019% | 0.010% | 0.008% | ***0.012%*** |
| Chloride channel (TC 2.A.49) | Firmicutes | 0.032% | 0.007% | 0.003% | 0.008% | 0.005% | ***0.011%*** |
| RecU | Firmicutes | 0.032% | 0.003% | 0.008% | 0.001% | 0.010% | ***0.011%*** |
| Kynureninase | Firmicutes | 0.016% | 0.000% | 0.015% | 0.002% | 0.018% | ***0.010%*** |
| Peptidase M24 | Firmicutes | 0.016% | 0.011% | 0.011% | 0.004% | 0.007% | ***0.010%*** |
| 2Fe2S Shethna-type ferredoxin | Firmicutes | 0.016% | 0.005% | 0.008% | 0.010% | 0.007% | ***0.009%*** |
| AzlD/HI_1737/HP1330 | Firmicutes | 0.016% | 0.008% | 0.012% | 0.005% | 0.004% | ***0.009%*** |
| Zinc-associated anti-sigma factor (ZAS) | Firmicutes | 0.016% | 0.003% | 0.004% | 0.001% | 0.019% | ***0.009%*** |
| Peptidase S15 | Firmicutes | 0.032% | 0.003% | 0.003% | 0.002% | 0.001% | ***0.008%*** |
| Bacterial beta-galactosidase small subunit | Firmicutes | 0.016% | 0.004% | 0.001% | 0.016% | 0.002% | ***0.008%*** |
| HFCD (homooligomeric flavin containing Cys decarboxylase) | Firmicutes | 0.032% | 0.002% | 0.000% | 0.004% | 0.000% | ***0.008%*** |
| UPF0251 | Firmicutes | 0.016% | 0.007% | 0.006% | 0.005% | 0.003% | ***0.007%*** |
| SorC transcriptional regulatory | Firmicutes | 0.016% | 0.002% | 0.008% | 0.002% | 0.008% | ***0.007%*** |
| Methylaspartate mutase GlmS subunit | Firmicutes | 0.016% | 0.006% | 0.003% | 0.008% | 0.002% | ***0.007%*** |
| Bacterial microcompartments protein | Firmicutes | 0.000% | 0.008% | 0.008% | 0.011% | 0.004% | ***0.006%*** |
| 3-oxoacid CoA-transferase | Firmicutes | 0.016% | 0.001% | 0.004% | 0.003% | 0.003% | ***0.005%*** |
| SMP-30/CGR1 | Firmicutes | 0.016% | 0.001% | 0.005% | 0.002% | 0.002% | ***0.005%*** |
| EutL/PduB | Firmicutes | 0.000% | 0.004% | 0.008% | 0.010% | 0.002% | ***0.005%*** |
| Glycosyl hydrolase 44 (cellulase J) | Firmicutes | 0.016% | 0.002% | 0.001% | 0.000% | 0.000% | ***0.004%*** |
| BtpA | Firmicutes | 0.016% | 0.000% | 0.001% | 0.001% | 0.001% | ***0.004%*** |
| UPF0754 | Firmicutes | 0.000% | 0.001% | 0.003% | 0.000% | 0.014% | ***0.004%*** |
| Fructosamine kinase | Firmicutes | 0.016% | 0.000% | 0.000% | 0.002% | 0.000% | ***0.004%*** |
| Oxoprolinase | Firmicutes | 0.000% | 0.002% | 0.004% | 0.003% | 0.002% | ***0.002%*** |
| YmdB | Firmicutes | 0.000% | 0.002% | 0.002% | 0.003% | 0.001% | ***0.002%*** |
| PhzF | Firmicutes | 0.000% | 0.002% | 0.003% | 0.000% | 0.003% | ***0.002%*** |
| Peptidase M4 | Firmicutes | 0.000% | 0.001% | 0.002% | 0.000% | 0.000% | ***0.001%*** |
| SarZ | Firmicutes | 0.000% | 0.000% | 0.001% | 0.001% | 0.001% | ***0.001%*** |
| Ornithine cyclodeaminase/mu-crystallin | Firmicutes | 0.000% | 0.001% | 0.001% | 0.000% | 0.001% | ***0.001%*** |
