# Supplementary material for: Diversity and functions of the sheep faecal microbiota: a multi‐omic characterization
Source: Microb Biotechnol. 2017 Feb 6;10(3):541–54. doi: 10.1111/1751-7915.12462 (PMC5404191; doi:10.1111/1751-7915.12462)
Supplement: Supplementary file 5 — Table S4. Glycosyl hydrolase families detected in all animals by shotgun metagenomics. [file MBT2-10-541-s005.docx]

**Table S4.** Glycosyl hydrolase families detected in all animals by shotgun metagenomics.

| **Gene family** | **Sheep 1** | **Sheep 2** | **Sheep 3** | **Sheep 4** | **Sheep 5** | ***Mean*** |
| --- | --- | --- | --- | --- | --- | --- |
| Glycosyl hydrolase 13 | 0.740% | 0.555% | 0.562% | 0.783% | 0.465% | ***0.621%*** |
| Glycosyl hydrolase 3 | 0.432% | 0.484% | 0.390% | 0.388% | 0.318% | ***0.402%*** |
| Glycosyl hydrolase 2 | 0.236% | 0.310% | 0.284% | 0.293% | 0.213% | ***0.267%*** |
| Glycosyl hydrolase 51 | 0.175% | 0.168% | 0.155% | 0.252% | 0.107% | ***0.171%*** |
| Glycosyl hydrolase 1 | 0.339% | 0.123% | 0.095% | 0.162% | 0.085% | ***0.161%*** |
| Glycosyl hydrolase 43 | 0.195% | 0.154% | 0.122% | 0.167% | 0.129% | ***0.154%*** |
| Glycosyl hydrolase 31 | 0.123% | 0.151% | 0.136% | 0.180% | 0.112% | ***0.140%*** |
| Glycosyl hydrolase 4 | 0.072% | 0.117% | 0.123% | 0.161% | 0.082% | ***0.111%*** |
| Glycosyl hydrolase 97 | 0.072% | 0.137% | 0.070% | 0.054% | 0.086% | ***0.084%*** |
| Glycosyl hydrolase 42 | 0.113% | 0.054% | 0.077% | 0.119% | 0.046% | ***0.082%*** |
| Glycosyl hydrolase 130 | 0.031% | 0.075% | 0.066% | 0.105% | 0.064% | ***0.068%*** |
| Glycosyl hydrolase 36 | 0.051% | 0.075% | 0.072% | 0.091% | 0.051% | ***0.068%*** |
| Glycosyl hydrolase 10 (cellulase F) | 0.062% | 0.081% | 0.061% | 0.063% | 0.053% | ***0.064%*** |
| Glycosyl hydrolase 5 (cellulase A) | 0.041% | 0.058% | 0.058% | 0.072% | 0.044% | ***0.055%*** |
| Glycosyl hydrolase 20 | 0.041% | 0.076% | 0.043% | 0.037% | 0.044% | ***0.048%*** |
| Glycosyl hydrolase 67 | 0.041% | 0.039% | 0.032% | 0.054% | 0.028% | ***0.039%*** |
| Glycosyl hydrolase 53 | 0.010% | 0.028% | 0.039% | 0.047% | 0.019% | ***0.029%*** |
| Glycosyl hydrolase 32 | 0.062% | 0.021% | 0.018% | 0.022% | 0.015% | ***0.028%*** |
| Glycosyl hydrolase 94 | 0.010% | 0.030% | 0.024% | 0.040% | 0.024% | ***0.026%*** |
| Glycosyl hydrolase 127 | 0.041% | 0.015% | 0.017% | 0.031% | 0.007% | ***0.022%*** |
| Glycosyl hydrolase 84 | 0.031% | 0.024% | 0.015% | 0.017% | 0.017% | ***0.021%*** |
| Glycosyl hydrolase 8 (cellulase D) | 0.021% | 0.021% | 0.016% | 0.031% | 0.012% | ***0.020%*** |
| Glycosyl hydrolase 65 | 0.010% | 0.008% | 0.022% | 0.033% | 0.011% | ***0.017%*** |
| Glycosyl hydrolase 27 | 0.041% | 0.010% | 0.012% | 0.010% | 0.007% | ***0.016%*** |
| Glycosyl hydrolase 39 | 0.021% | 0.007% | 0.011% | 0.025% | 0.007% | ***0.014%*** |
| Glycosyl hydrolase 26 | 0.010% | 0.011% | 0.011% | 0.013% | 0.005% | ***0.010%*** |
| Glycosyl hydrolase 11 (cellulase G) | 0.010% | 0.008% | 0.011% | 0.003% | 0.007% | ***0.008%*** |
| Glycosyl hydrolase 44 (cellulase J) | 0.010% | 0.002% | 0.001% | 0.001% | 0.000% | ***0.003%*** |
